# T Helper (Th) Cell Profiles in Pregnancy and Recurrent Pregnancy Losses: Th1/Th2/Th9/Th17/Th22/Tfh Cells

**DOI:** 10.3389/fimmu.2020.02025

**Published:** 2020-08-18

**Authors:** Wenjuan Wang, Nayoung Sung, Alice Gilman-Sachs, Joanne Kwak-Kim

**Affiliations:** ^1^Reproductive Medicine and Immunology, Obstetrics and Gynecology, Department of Clinical Sciences, Chicago Medical School, Rosalind Franklin University of Medicine and Science, North Chicago, IL, United States; ^2^Reproductive Medicine Center, The Affiliated Yantai Yuhuangding Hospital of Qingdao University, Yantai, China; ^3^Center for Cancer Cell Biology, Immunology and Infection, Chicago Medical School, Rosalind Franklin University of Medicine and Science, North Chicago, IL, United States; ^4^Clinical Immunology Laboratory, Center for Cancer Cell Biology, Immunology and Infection, Chicago Medical School, Rosalind Franklin University of Medicine and Science, North Chicago, IL, United States

**Keywords:** Th1 cell, Th2 cells, Th9 cells, Th17 cells, Th22 cells, pregnancy, recurrent pregnancy loss

## Abstract

During pregnancy, various immune effectors and molecules participating in the immune-microenvironment establish specific maternal tolerance toward the semi-allogeneic fetus. Activated maternal immune effectors by the trophoblast antigens, such as T helper (Th), T cytotoxic (Tc), T regulatory (Treg), and B cells, are involved in the regulation of adaptive immunity. Recognition of active signal through the T cell receptors stimulate the differentiation of naive CD3^+^CD4^+^ T cells into specific T cell subsets, such as Th1, Th2, Th9, Th17, Th22, and follicular Th cells (Tfh). Each of these subsets has a significant and distinct role in human pregnancy. Th1 immunity, characterized by immune-inflammatory responses, becomes dominant during the peri-implantation period, and the “controlled” Th1 immunity benefits the invading trophoblasts rather than harm. Quickly after the placental implantation, the early inflammatory Th1 immunity is shifted to the Th2 anti-inflammatory immune responses. The predominant Th2 immunity, which overrules the Th1 immunity at the placental implantation site, protects a fetus by balancing Th1 immunity and accommodate fetal and placental development. Moreover, Treg and Th9 cells regulate local inflammatory immune responses, potentially detrimental to the fetus. Th17 cells induce protective immunity against extracellular microbes during pregnancy. However, excessive Th17 immunity may induce uncontrolled neutrophil infiltration at the maternal-fetal interface. Other Th cell subsets such as Tfh cells, also contribute to pregnancy by setting up favorable humoral immunity during pregnancy. However, dysregulation of Th cell immunity during pregnancy may result in obstetrical complications, such as recurrent pregnancy losses (RPL) and preeclampsia (PE). With this review, we intend to deliver a comprehensive overview of CD4^+^ Th cell subsets, including Th1, Th2, Th9, Th17, Th22, and Tfh cells, in human pregnancy by reviewing their roles in normal and pathological pregnancies.

## Introduction

T helper (Th) dichotomy of Th1/Th2 immune responses was proposed by Mosmann and colleagues (1) in 1986. This concept was adapted to explain maternal tolerance toward fetal alloantigen in 1993 by Wegmann et al. (2). Domination of Th2 immunity, which overrules Th1 immunity during pregnancy, was hypothesized to protect the fetoplacental unit from the attack of maternal Th1 cells (2). Indeed, obstetrical complications, for instance, recurrent pregnancy losses (RPL) and preeclampsia (PE), have been documented to be associated with predominant Th1 immunity (3–5), although RPL was also documented with shifted Th2 immunity (6, 7). The paradigm of Th1 and Th2 dichotomy was then incorporated with a concept of regulatory T cell (Treg) immunity, which was first described in 1979 by Chaouat and Voisin (8), to explain maternal-fetal tolerance further. In 2010, the Th1/Th2 paradigm for maternal immune tolerance was expanded to the Th1/Th2/Th17 and Treg cell paradigm (9). This paradigm was able to better answer the conundrum of fetal tolerance during pregnancy comprehensively by adopting not only Th17 immunity but Treg immunity, which confer expanded tolerance to encompass semi-allogeneic fetal antigens in addition to tolerance to self-antigen and extended-self commensal antigens (10).

Recent advances in immunology have revealed the existence of additional Th cell subsets at the maternal-fetal junction, of which roles beyond the contribution of Th1, Th2, and Th17 cells (11), including Th9 (12), Th22 (12–14), and follicular Th (Tfh) cells (15). In this review, we recapitulate the reported functions of various Th cells at the maternal-fetal junction and document how aberrant Th immunity is associated with RPL.

## Placental HLA Antigens Regulate T Cell Activation at Maternal-Fetal Junction

Villous trophoblasts, including cytotrophoblasts and syncytiotrophoblasts, and syncytiotrophoblast microparticles (STBM) lack any human leukocyte antigen (HLA) expression and do not present major histocompatibility complex (MHC) class I and II transcriptional activators, like class II transactivator and NOD-like receptor family CARD domain containing 5 (NLRC5) (16). CD3^+^CD4^+^ Th and CD3^+^CD8^+^ T cytotoxic (Tc) cells interplay with HLA class II and I antigens, respectively. Once activated, Th cells release various cytokines that are essential for the activation of Tc cells, isotype switching of B cells, and bactericidal activity of phagocytes, including macrophages. Therefore, lack of HLA class I and II antigens may protect villous trophoblast cells from T cell alloreactivity in normal pregnancy (17). However, in pathological pregnancies, such as PE, HLA-DR, HLA class II antigen, was detected in a significant proportion of syncytiotrophoblast and syncytiotrophoblast-derived extracellular vesicles (STEVs) that are either the fetal or maternal origin, possibly acquired by trogocytosis. Therefore, the activation of inflammatory Th cells in patients with PE may partly be explained by the pathological expression of HLA-DR in syncytiotrophoblast and STEV (18).

Contrary to villous trophoblast, HLA C, classical MHC class I molecule, HLA E, F, and G (19), non-classical MHC class I molecules, and MHC transcriptional activators, such as NLR family pyrin domain containing 2 (NLRP2), are expressed in extravillous cytotrophoblast (EVT) (16). NLRP2, highly expressed in EVT, is also expressed by the JEG3 human choriocarcinoma cells, which is the EVT cell line, but not in decidual stromal cells. Although NLRP2 is an MHC transcriptional activator, it has been reported to suppress constitutive HLA-C expression in JEG3 cells and TNF-α-induced NF-κB activation (16). Therefore, NLR family members may link innate immunity to adaptive immunity by participating in the regulation of MHC expression during inflammation. Since NLRP2 suppresses pro-inflammatory immune responses, it may contribute to avoid undesirable anti-fetal responses (16).

Maternal Tc lymphocytes recognize paternal HLA-C and minor histocompatibility antigens (mHAg), such as HY, on trophoblasts, which has been reported in numerous people and during a healthy pregnancy. Indeed, HLA-C histoincompatibility at the maternal-fetal junction has been documented to induce a tolerogenic microenvironment (20, 21). CD8^+^ Tc cells can indirectly recognize fetal antigen and undertake clonal deletion, yet, cytotoxic effector function is not primed; consequently, antigen-specific fetal demise cannot be induced even with artificial activation of these cells (22). Therefore, maternal T cells, particularly Tc cells, target EVT since it expresses paternal HLA-C, but its level of expression may determine how the maternal alloreactivity by Tc cells is molded. Tight transcriptional regulation of HLA-C expression allows balancing between the induction of tolerance and alloimmunity at the maternal-fetal junction (23). In mice, alloimmune recognition of maternal T cells was exclusively through maternal antigen-presenting cells (APCs), which uptake and process the fetal antigens. Contrarily, in the human placenta, T cell recognition of allogeneic fetus was severely restricted, since antigen presentation is constrained by low levels of antigen uptake, and gradual presentation of fetal antigens to T cells, via maternal APCs, such as dendritic cells (DCs), allowing immune tolerance (22, 24). Once activated, CD4^+^ Th cells participate in maternal immune responses, particularly by regulating adaptive immunity (22). When decidual DCs were specifically exhausted, the poor decidualization of human endometrium was noticed with inadequate endometrial vascularity, leading to impaired implantation of blastocysts (25). Therefore, not only the characteristics of placental HLA antigen expression but its presentation via APC may participate in T cell regulation at the maternal-fetal interface and inducing maternal tolerance to fetal antigens.

## Th1 Immunity for Controlled Inflammation During Implantation

A propensity to Th1 over Th2 immune response is generally recognized during the peri-implantation period by the presence of immune-inflammatory changes. The controlled Th1 shift actually benefits the invading trophoblasts rather than damaging them (26). During early pregnancy, the inflammatory priming of peripheral blood mononuclear cells (PBMCs) is established (26). Circulating STBM stimulates the various inflammatory cytokine production, including IL-12, TNF-α and low level of IL-18, from monocytes (27) and contribute to establishing a mild systemic inflammation. Consequently, low IL-18 level induces limited production of IFN-γ, by which an overall type 2 immunity is maintained during pregnancy (26, 28). PBMCs can be recruited to decidua. Specifically, chemokine receptor expressions (especially CCR molecules) on CD4^+^ T cells determine their trafficking patterns, including the target tissue, timing, and signals to receive (29). Once T cells are recruited to the uterine lining, these cells are induced to display unique phenotypic properties by the micromilieu of the maternal-fetal interface (30, 31). Quickly after the placental implantation, the Th2 shift is seemingly noticeable, which is critical for the maintenance and development of normal fetus and placenta (32), and as well as suppression of Th1 immunity at the maternal-fetal junction (2, 9, 13, 33).

Th1 cells secrete various cytokines, typically interleukin (IL)-2, TNF-α, and IFN-γ, which participate in immune surveillance and prevent excessive trophoblast invasion (34). TNF-α has been reported to protect the fetoplacental unit (34) and have a regulatory role in trophoblast invasion by altering trophoblast cell adhesion to laminin and inhibiting trophoblast cell mobility *in vitro* (35). Contrarily, TNF-α has been associated with the immunopathology of various obstetrical complications. TNF-α elevates trophoblast-derived plasminogen activator inhibitor-1 (PAI-1) levels and decreases the invasive capacity of trophoblasts (36, 37). TNF-α produced by monocytes from preeclamptic patients induces apoptosis of human trophoblast cells (38) and inhibits JEG-3 (trophoblast-like cell) incorporation into maternal endothelial cell complex by inhibiting MMP-2 and aborting integrin switch from α6β4 to α1β1. TNF-α activates endothelial cells (38, 39), and activated monolayer endothelial cells repel JAR cell incorporation (40). TNF-α induces matrix metalloproteinases-9 (MMP-9) but not MMP-2 expression in the decidua of preeclamptic women and disrupts the decidual extracellular matrix to interfere with normal stepwise EVT invasion (41, 42). Therefore, a delicate balance of TNF-α at the placentation site is critical for a successful pregnancy.

IFN-γ mRNA expression has been reported in implantation sites of healthy pregnant women and the murine model (43, 44). IFN-γ has an essential role in vascular remodeling during the peri-implantation period (45, 46). In mice, the local IFN-γ levels of the pregnant uterus reached a peak on gestation day (GD) 10, which was significantly higher than the baseline IFN-γ level (47). IFN-γ increases EVT apoptosis and/or decreases protease activity, in turn, regulating EVT invasion. Hence, IFN-γ has a critical role in early placentation and the trophoblast invasion process. Contrary to these physiological roles, IFN-γ has a potent pro-inflammatory role. It increases HLA class I and II antigen and toll-like receptor (TLR) expressions in innate immune cells, promotes isotype commutation, induces chemokine secretion, activates macrophages, and increases phagocytosis (48). In a porcine model, increased IFNG gene expression at the placental attachment site was associated with early arresting conceptus on gestation day (GD) 20, while the site of a late arresting conceptus (GD 50) had increased TNF mRNA expression (49), suggesting a presence of specific localization mechanism of cytokine expression regulated by the fetal placental unit and phase-specific cytokine responses during pregnancy (50).

The potential immunopathological effects of type 1 cytokines on pregnancy have been demonstrated in animal studies and human pregnancies. Lipopolysaccharide (LPS) injection to 14.5 gd pregnant Wistar rats induced maternal inflammation and subsequent fetal losses in a dose-dependent manner. Alive fetuses had significant growth restriction as well. Administration of IL-10, which has immunoregulatory properties, and TNF-α receptor blocker etanercept, prevented LPS-induced pregnancy losses (51). In addition, either the direct introduction of Th1-type cytokines in large amounts, such as IL-2 or IFN-γ or indirect increase of Th1-type cytokines by activation of TLR induced fetal resorption in mice (52). In human pregnancy, increased percentages of IFN-γ^+^/Th1 and IFN-γ^+^/Tc1 cells were reported in the decidua of women who miscarried a genetically normal fetus (*N* = 19) as compared with those of induced abortions (*n* = 15) (53). In addition, decidual T cells from women with miscarriage expressed increased IL-2 and IFN-γ, and decreased IL-4 and IL-10 as compared with those of normal pregnancy, and Th1 cytokine production was positively correlated with CD86 and CD28 expression (54). In women with RPL, Th1 bias was present both locally (decidua) and systemically at the time of abortion (*N* = 24) as compared to those at the time of normal delivery (*N* = 39) (55). In a study of women who experienced RPL (*N* = 26), IFN-γ^+^/IL-4^+^, TNF-α^+^/IL-4^+^, and TNF-α^+^/IL-10^+^ producing Th1/Th2 cell ratios were significantly higher in the peripheral blood than those of healthy fertile controls (56). Additionally, women with RPL had increased T cell activation with systemic Th1 dominance compared to normal fertile women (57). When shifted Th1 immunity was regulated with intravenous immunoglobulin G (IVIg), TNF blockers, or T cell activation inhibitors, such as etanercept, adalimumab, or tacrolimus, pregnancy outcome was significantly improved in women with RPL (58–62). Therefore, time and cytokine-specific regulation of Th1 immunity during pregnancy is critical for a successful pregnancy outcome.

Underlying autoimmune diseases may contribute to Th1 immunity during implantation and pregnancy. In autoimmune diseases, including Hashimoto’s thyroiditis (63), systemic lupus erythematosus (64), antiphospholipid antibody syndrome (65), the initial stage of Sjogren’s syndrome (66) and scleroderma (67), shifted Th1 immunity or Th17 upregulation have been reported, and RPL has been often associated with these conditions (68–70). For instance, in women with Hashimoto’s thyroiditis, Th1 cells actively involve the inflammatory lymphocyte infiltration of the exocrine glands and epithelia and cause subsequent thyroiditis, glandular damages, and apoptosis of thyroid cells (71). Moreover, Th1 cells are also recruited to the endometrium, decidua, and placenta, and activate NK cells, resulting in spontaneous abortion (72). Therefore, Th1 immunity, a shared underlying immunopathology, induces RPL in women with autoimmune diseases.

## Th2 Immunity and Maternal-Fetal Tolerance

Th2 immune dominance has been documented in both normal pregnancy (1, 73) and RPL (7, 9, 74). During pregnancy, uterine DCs actively participate in the naive T cell differentiation into Th2 cells (75). Paternal antigens in trophoblasts activate Th2 cells at the maternal-fetal junction (3, 76, 77) and Th2 cells infiltrate into the decidua basalis (78, 79). Th2 cells, then, induce local Th2 dominance by releasing Th2-type cytokines, which could promote maternal-fetal tolerance. In a rodent study, when Th2 immunity was induced by IL-10 administration to a pregnant mother, pregnancy loss was prevented (51, 80). Th2 immunity represses the development of Th1 and Th17 immunities by releasing IL-4 and IL-13, respectively, and promotes allograft tolerance (81). Therefore, IL-10 or another type 2 cytokines may improve pregnancy success (82).

As pregnancy progresses further, maternal immune responses are shifted away from type 2 immunity toward inflammatory and counterregulatory responses. In a study analyzing serum from pregnant women (*n* = 16), IL-1β, IL-6, IL-8, IL-12p70, IL13, IL-15, IP-10, and FLT3-ligand levels were increased in relation to gestational weeks while serum IFNα2, IL-1ra, IL-3, IL-9, IL-12p40, and soluble CD40L levels were increased with the advancement of the trimester (83). The re-shifting of Th2 immunity to inflammatory responses may be associated with the preparation of parturition.

Th2 cells may participate in autoantibody production and enhance autoimmunity. The aberrant and sustained expression of IL-4 *in vivo* prolonged autoreactive B cell survival by suppressing apoptosis. Th2 cytokine, IL-4, was reported to induce autoreactive B cell activation and thus promotes autoimmunity (84). Therefore, exacerbated Th2 immunity during pregnancy may induce autoimmune diseases or flare-up underlying autoimmune conditions. Th2-type autoimmune diseases, such as systemic lupus erythematosus, could get worse (85) when type 2 cytokines were increased during pregnancy. Additionally, increased tolerogenic signals from Th2 cells may induce uncontrolled viral infections. In the fatal ZIKA virus-induced microcephaly, predominant expression of Th2 cytokines was found in meninges, perivascular space, and parenchyma. It was speculated that the active participation of Th2 cells was the immunopathogenic mechanisms of the ZIKA virus (47). Hence, timely and adequate Th2 immunity is important for the immunotolerance and protection of fetus from infection.

## Increased Tfh Cells During Pregnancy to Favor Humoral Immunity

Follicular Th cells are characteristically recognized by the master regulator transcription factor, B cell 6 (BCL6), CXCR5, and STAT3 (86). Tfh cells offer cognate assistance to B cells (87). Pregnant women in the third trimester have a higher proportion of peripheral blood Tfh cells (%) than non-pregnant women, despite co-expressing markers, including programmed death (PD)-1, ICOS, or CXCR3. Furthermore, pregnant women had a significantly higher proportion of CXCR3^+^ Tfh cells (%) than non-pregnant women, which can secrete IL-6, IL-10, and IL-21, and notably, PD-1^+^/CXCR3^+^ Tfh cells are positively associated with peripheral blood estrogen levels (15). In mice, uterine and placental CD4^+^ T cells express CD4^+^CXCR5^*hi*^PD-1^*hi*^ and CD4^+^CXCR5^*hi*^ICOS^*hi*^ phenotypes like Tfh cells, which represent memory and activation status. In the uterus, these cells are abundant and reach a peak at mid-gestation; however, in the placenta, they are increased at late gestation (88). Pregnancy, which accompanies the high estrogen level, has been suggested to positively influence the proportion of Tfh cells specialized in helping B cells, thus, favoring humoral immunity (15) and balancing Th1/Th2 immune responses. Therefore, during pregnancy, a timely and proper accumulation of Tfh cells may assist in the maintenance of a successful pregnancy, while an excessive or lack of accumulation could result in miscarriage (88).

## The Role of Th9 Cells in the Progress of Parturition

Th9 cells, which are determined by potent production of IL-9, might participate in host-pathogen interaction, anti-tumor immunity, and immune-mediated diseases, including allergy and autoimmune inflammatory diseases (89). Th9 cells, a subpopulation of Th2 cells, are different from Th2 cells phenotypically and functionally, and subject to PPAR-γ (90). TGF-β can convert Th2 cells to IL-9 producing cells (91). Furthermore, Th1 or Th17 cell fostering cytokines like IL-1β, IL-12, or IL-21, enhance IL-4/TGF-β-induced CD4^+^IL-9^+^ T cell differentiation when pbCD3/sCD28 stimulation is present (92). Although IL-9 has been historically categorized by type 2 cytokines (93), innate lymphoid cells (ILC) mostly produce IL-9 (94). Th-17 cells produce IL-9 when TGF-β is present, and IL-9 participates in the induction of inflammation (95). During pregnancy, peripheral blood IL-9 levels are increased as a function of the trimester, plausibly being resultant of changes in the cellular composition of peripheral blood (83). In a mouse study, IL-9 was reported to present in the non-pregnant uterus. During pregnancy, it remained at a high level in both uterus and placenta, signifying its regulatory role in local inflammatory immune responses which can be a potential threat to the offspring (96). In mice, the decline in decidual Th9 cells together with IL-6 controlled infiltration of CD4^+^ T and CD8^+^ Treg cells in decidua were involved with progress of parturition (97). Studies for Th9 cells in human pregnancy are limited currently, and a possible role of Th9 cells in parturition should be investigated further.

## Aberrant Th17 Immunity in Pregnancy Losses

In peripheral blood of healthy population, Th17 cells are rare among CD4^+^ T cells (0.64–1.4%) (98, 99). During all stages of pregnancy, Th17 cell proportions among CD4^+^ T cells were reported to be comparable to that of non-pregnant status (99), although another study reported that the pregnant women in the third trimester had a decreased proportion of Th17 cells as compared with non-pregnant women (100). The decidua contains a higher density of Th17 cells than in the peripheral blood (99), and the decidual IL-17^+^ cell count was parallel to the neutrophil count, which suggests that IL-17^+^ cells are intimately involved in neutrophil infiltration (101) and induce protective immunity against extracellular microbes in the uterus. Th17 cells are not only involved with infectious immunity but contribute to human pregnancy. In a study of JEG3 cells, the capacities of progesterone secretion and tissue invasion were increased with IL-17 stimulation (102). Additionally, Th17 cells induce activation of decidual NK (dNK) cells and impair vascular reactivity of uterine arteries (103), leading to embryo resorption (103–105). Excessive Th17 cells have been detected in the decidua and peripheral blood of inevitable abortion (11, 106–110). IL-7/IL-7R pathway was reported to up-regulate Th17 immunity while downregulating Treg immunity (111).

Women with unexplained RPL (uRPL) demonstrated an increased proportion (%) of peripheral blood Th17 cells and related cytokine levels (IL-17A) during the proliferative and secretory phases of the ovulatory cycle than those of normal controls (112). Contrarily, Treg cells and associated cytokines, including IL-10 and TGF-β1, were lower during the proliferative phase in women with uRPL than those of controls. Women with uRPL had a significantly higher Th17/CD4^+^ Treg cell ratios as compared to fertile controls (107), during both proliferative and secretory phases (112). Hence, the balance between Th17/Treg cell immunity may determine pregnancy outcome.

## Promotion of Trophoblast Survival by Th22 Cells

Th22 cells typically produce IL-22 deprived of IL-4, IFN-γ, and IL-17, and the primary transcription molecule for the Th22 cell differentiation is the aryl hydrocarbon receptor (AHR) (113). IL-22 is involved in various physiologic and pathologic processes, such as immune regulation and allograft rejection, respectively. Hence, IL-22 could potentially harm a pregnancy. At the maternal-fetal junction, the proliferation and viability of trophoblast cells were promoted by IL-22, and apoptosis was significantly reduced by IL-22 (114). IL-22 plays a critical role in protecting trophoblast cells from pathogens (115) and inflammatory immune responses following intrauterine infection (116). Indeed, IL-22 receptors (IL-22R) are expressed on placental villi, and IL-22 from decidual stromal cells and dNK cells bind to the IL-22R1, a subunit of IL-22R (114). IL-22/IL-22R1 pathway might be involved in the promotion of trophoblasts survival and pregnancy maintenance. Women with a history of uRPL demonstrate lower IL-22R1 expression compared to women with normal pregnancy (114). Hence, the aberrant activation of IL-22/IL-22R1 pathways in placental villi might be linked to the manifestation of spontaneous abortion (114).

RORγt and T-bet regulate Th22 differentiation positively and negatively, respectively, from naïve Th cells. Conversely, Th22 cells can be differentiated into Th1 or Th2 cells. Th22 cells demonstrated marked plasticity to produce IFN-γ in Th1 promoting conditions *in vitro* or IFN-γ-rich inflammatory micromilieu *in vivo* (117). Th22 cells also demonstrate plasticity to increase IL-13 secretion under the Th2 environment *in vitro* (117). Decidual CD4^+^ T cells secreted a more substantial amount of IL-22 than peripheral blood CD4^+^ T cells, regardless of normal pregnancy or uRPL (14). Women with uRPL have significantly higher IL-17, IL-21, and IL-22, and significantly lower TGF-β serum concentrations than those of normal controls (118). However, the contradictory result was also reported in women with uRPL with a euploid fetal loss that significantly lower IL-22 gene expression in the decidua, and peripheral blood was demonstrated than normal pregnancy (119).

**FIGURE 1:**
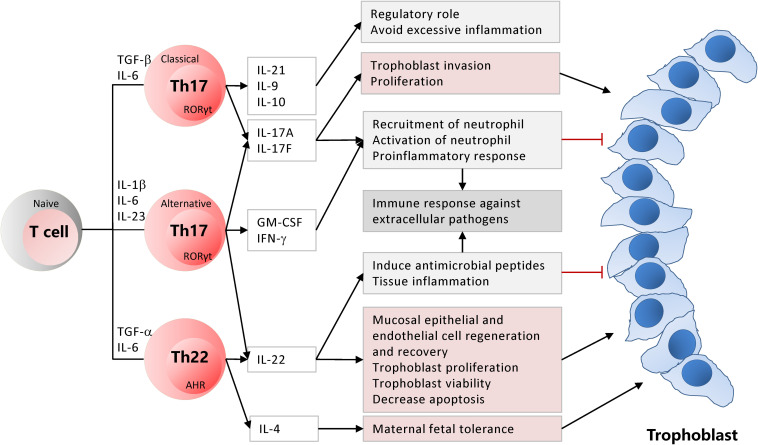
Classical and alternative Th17 and Th22 subsets are differentiated from the naïve CD4^+^ T cells based on differentiation cytokines. Activated Th17 and Th22 cells have critical roles for the maintenance of pregnancy while establishing inflammation and immunity against extracellular pathogens at the maternal-fetal junction.

In a successful pregnancy, IL-22 and IL-4 producing CD4^+^ T cells, i.e., Th17/Th2, Th17/Th0, and Th0 subsets, were predominant in the decidua. Contrarily, IL-22 only secreting CD4^+^ T cells, i.e., Th17/Th1 subset, were prevalent in the decidua from uRPL (14). In a successful pregnancy, IL-22^+^ Th17/Th2 and Th17/Th0 subsets mainly existed, and GATA-3, ROR-C, AHR, IL-4, IL-17A, and IL-22 mRNA expressions were detected at the implantation site. While mRNA expression of T-bet and IFN-γ was detected mainly away from the implantation site. Therefore, IL-22^+^ Th17/Th2 and Th17/Th0 subsets may have a critical role in embryo implantation (14) (Figure 1). Whether the decidual IL-22 expression is associated with uRPL has not been explored yet.

## The Regulation of T Helper Cells via Co-Inhibitory Receptors

T helper cell activation and differentiation involve genetic control of gene expression by the concerted mechanism of various cytokines, transcription factors, and epigenetic regulators (120). PD-1 (CD279) is a transmembrane inhibitory receptor that ligates with PD-L1 or PD-L2 (121). After stimulation, lymphocytes in various lymphoid organs and tissues with ongoing immunity express PD-1 (122). PD-1/PD-L1 (CD274) axis participates in suppressing autoreactive immune effectors and, consequently, achieve T cell homeostasis. Accordingly, the PD-1/PD-L1 axis may promote to establish maternal-fetal tolerance (123, 124).

PD-1/PD-L1 axis participates in T cell regulation by suppressing T cell activation and differentiation, modifying molecular secretion patterns, and inducing cell death and exhaustion of T cells (125–127). The downstream outcome of the PD-1/PD-L1 axis includes the inactivation of PI3K/AKT and Ras-MEK-ERK signaling pathways (128). Anti-PD-1 and anti-PD-L1 block this pathway and induce Tregs suppression. Additionally, these molecules accelerate T cell proliferation and infiltration, disrupt T cell equilibrium, and induce pro-inflammatory micromilieu over tolerance at the implantation site (129). In mice, the PD-1/PD-L1 axis blockade reduced the Treg cells, which result in pregnancy losses by inducing Th1 and Th17 cell differentiation (124, 130, 131). PD1/PD-L1 pathway can also suppress Th22 and Th9 cells through negative costimulatory interactions (132, 133). In the peripheral blood of women with RPL, CD4^+^ T cells with IFN-γ^+^/CD279^+^ and TNF-α^+^/CD279^+^ (PD-1^+^ Th1) and IL17^+^/CD279^+^ (PD-1^+^ Th17) immunophenotypes were significantly lower than those of fertile controls. Women with RPL have significantly lower PD-L1^+^ Th17 cells, but the similar proportions of PD-L1^+^ Th1 and Treg cells than those of controls (134). Therefore, the propensity to Th1 and Th17 immunity may be associated with RPL by down-regulation of PD-1 expression and a sequential disequilibrium among Th17, Th1, and Treg cells (134).

Initially, the T cell immunoglobulin domain and mucin domain (Tim)-3 was recognized as a Th1-specific receptor present on the cell surface. It transduces an apoptotic signal by engaging Gal-9 and suppresses Th1 immunity (135, 136). Tim-3 and its ligand Gal-9 interaction initiates intracellular calcium influx and suppresses Th1 and Th17 cells (137–139). The Tim-3 blockade induced increased Th1 cytokines at the maternal-fetal junction, and immune tolerance was disrupted (140). Contrarily, Tim-3 can also keep decidual stromal cells from inflammatory damages and TLR-mediated apoptosis by enhancing Th2 immunity at the maternal-fetal junction (141, 142). Thus, in TLR-triggered inflammation during pregnancy, Tim-3 signaling may function as a self-control mechanism (141). The Tim-3 expression was significantly up-regulated in villi and deciduas of pregnant women with RPL compared to normal pregnancies (142–144) as well as in abortion-prone matings in animal models (145, 146), representing that the Tim-3 molecule might participate in the immunopathology of RPL (143, 145, 147–149).

Decidual CD4^+^ T cells may be controlled by PD-1 and Tim-3, by which tolerance at the implantation site is promoted. In a normal pregnancy, the decidua contains a higher count of PD-1^+^/Tim-3^+^ CD4^+^ T cells than peripheral blood, suggesting their role to support Th2 immunity at the implantation site (135). Contrarily, pregnancy losses may be associated with decreased decidual PD-1^+^/Tim-3^+^ CD4^+^ T cells. However, regardless of pregnancy outcome, Th1 cytokine secretion from these cells was not changed. It indirectly meant that during pregnancy, PD-1 or Tim-3 molecules did not negatively regulate Th1-mediated immunity (149). Instead, the Th2 cytokines from decidual CD4^+^ T cells were promoted at the implantation site when both PD-1 and Tim-3 pathways were targeted together in a mouse model (149).

## Phase-Specific Th1/Th2 Balance in a Successful Pregnancy

**FIGURE 2:**
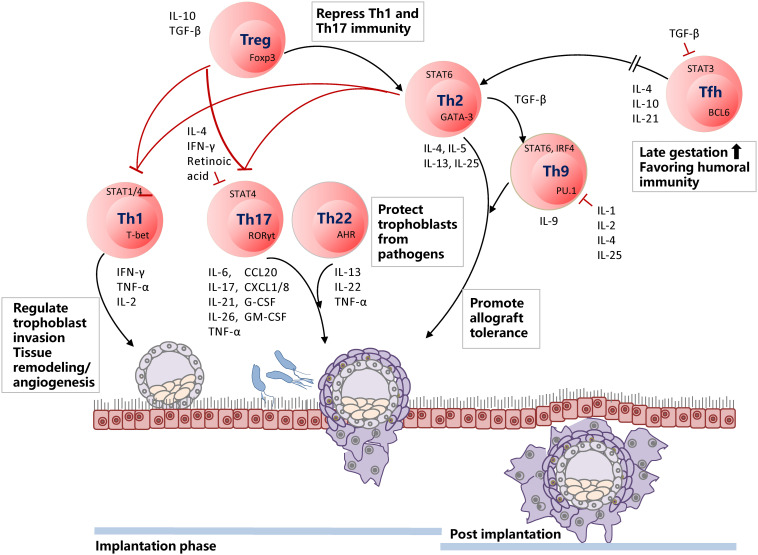
Timely shifting of Th1/Th2 and Treg/Th17 balance is of utmost importance for a successful pregnancy. During the implantation phase, Th1 cells are infiltrated at the decidua and regulate trophoblast invasion by participating in tissue remodeling and angiogenesis. Th17 and Th22 cells are present in the decidua but not enriched. Th17 cells involve in neutrophil infiltration and induction of protective immunity against extracellular microbes in the uterus. Th22 cells also protect trophoblast cells from pathogens and inflammatory immune responses from intrauterine infection. Decreased Th22 cells induce dysfunction of decidual stromal cells and NK cells. Hence, Th22 cells are keenly associated with Th1/Th2 balance. Treg and Th2 cells repress excessive Th1 and Th17 immunity. When the implantation phase is over, Th2 and Th9 cells become dominant and promote allograft tolerance. Tfh cells are usually enriched at late gestation, favoring humoral immunity and, thus, balancing Th1/Th2 immune responses.

Cytokine production is modified to prepare for the implantation and continuously adapted during pregnancy in many immune cells, including CD4^+^ Th cells (26, 27, 50). During the window of implantation, IFN-γ related gene expressions, such as IL-15, in human endometrium are elevated, but not IFNG (150). IL-15, responsible for NK cell proliferation and survival, activates CD56^*bright*^CD16^–^ uNK cells, which play a role in early INF-γ expression (151). During implantation, moderate infiltration of decidual Th1 cells is present, but Th2 and Th17 cell densities are not increased (152). Treg and Th2 cells promote allograft tolerance by repressing Th1 and Th17 cells, while Th17 and Th22 cells protect trophoblasts from pathogens (Figure 2).

Pro-inflammatory Th1-immune response is necessary at the time of implantation to promote tissue remodeling and angiogenesis (153). However, uncontrolled Th1 or Th17 immunity has been related to implantation failures, early pregnancy losses, and repeated implantation failures (56, 108). Several factors may affect the Th1 and Th2 balance at the placental implantation site. The local changes in Treg or NKT cells may induce Th1 propensity with up-regulated IFN-γ and down-regulated IL-4, and IL-10 expressions, which in sequence, results in RPL (11, 154, 155). Estrogen and testosterone have been shown to influence cytokine production from T cells and participate in the pathogenesis of RPL (156). Contrarily, progesterone can actively induce IL-4, when its concentration reaches the same level present at the feto-maternal junction (33, 76, 157). Multiple genes and cytokines expressed in the decidua play an essential role in protecting an invading embryo against excessive Th1 immunity, including TNF weak inducer of apoptosis (TWEAK), its receptor Fn-14, and IL-18, a Th2 promoting cytokine (158).

Once the implantation phase is over, Th1 immune dominance is gradually shifted to Th2 dominance (Figure 2). Decreased number of IL-12 producing decidual DC cells and myeloid DCs induce naïve CD4^+^ T cells to differentiate more into Th2 cells, contributing Th2 cell maintenance in the decidua (46). Moreover, increased Tfh cells during pregnancy favor Th2 immunity, thus balancing Th1/Th2 immunity (15). Activated T cells express galectin-1, which induces down-stream apoptosis (159), repressing Th1 immunity. Galectin-9 (Gal-9), which belongs to the β-galactoside-binding lectin family, participates in the regulation of Th1/Th2 balance via suppressing the production of Th1 cytokine, TNF-α, and promoting the secretion of Th2 type cytokine, IL-4 (160). Lastly, during pregnancy, major immunomodulatory molecules from the trophoblast, for example, HLA-G5 (161), favor a shift to Th2 immunity, which may participate in the upkeep of maternal tolerance to fetal antigens (77).

In plasma or serum from normal pregnant women, IL-18 elevation lasts only in the first trimester, IL-12p70 elevation is detected during the first and second trimester, and TNF production is steadily elevated throughout normal pregnancy (26). Once the implantation is over, Th1 immunity is quickly shifted toward the anti-inflammatory Th2 immunity, which dominates until the parturition. NK cells start shifting to Th2 phenotypes during the first trimester by themselves or their products (27), followed by other immune effectors. At the time of parturition, “the second” pro-inflammatory Th1 immunity appears to prepare for the labor and delivery (162, 163). Therefore, the timely shift and adequate balance between Th1 and Th2 immune responses during pregnancy are seemingly critical for a successful pregnancy. Contrarily, failure to achieve a proper balance during pregnancy is associated with obstetrical complications. Increased peripheral blood Th1/Th2 cell ratios have been reported in patients with RPL (56, 164) and preeclampsia (5).

## The Reciprocal *Trans-*Differentiation Between Th1, Th2, and Th17 Cells

*Trans-*differentiation of immune effectors makes the more complicated scenario of immune responses at the maternal-fetal junction. Th17 precursors can differentiate into the Th17/Th1 subset which secrets both IL-17 and IFN-γ, and ultimately differentiate into Th1 cells by IL-12 in the microenvironment (165). Whereas memory Th17 cells may differentiate into the Th17/Th2 subset when IL-4 is abundant in the microenvironment, and Th17/Th2 cells typically secrete IL-17 and IL-4 (166, 167). The majority of decidual CD4^+^ T cells produce both IL-4 and IL-17, so-called Th17/Th2 subset, at the embryo implantation site during successful pregnancy. Contrarily, pro-inflammatory Th17/Th1 cells are prevalent away from the implantation site of an embryo in a successful pregnancy and unexplained RPL (161). In humans, embryo and trophoblast cells produce soluble HLA-G5, which initiates Th17 cell differentiation into Th17/Th2 cells (161). Therefore, decidual micromilieu and HLA products actively control Th cell subsets at the maternal-fetal interface.

## Therapeutic Approaches to Modulate T Helper Cell Responses

T helper cells and their specific subsets could be utilized for diagnostic and therapeutic targets. In women with RPL, RIF, and autoimmune diseases, Th1/Th2 and Th17/Treg cell ratios in peripheral blood are significantly increased as compared to those of fertile controls (56, 71, 108). Additionally, abortion prone women with successful pregnancy had type 2 cytokine dominance in peripheral blood than abortion prone women with miscarriage (55). Based on these findings, Th cell-related biomarkers, such as Th1/Th2 cell ratios and TNF-α^+^ Th1 cell levels, have been translated to clinical medicine, and the diagnostic values of those biomarkers were determined by receiver operating characteristic curve analysis, which was specific to RPL (168). However, the verification of other biomarkers, such as specific cytokines, Th17/Treg ratios, Th17, and Treg levels, has not been made yet in women with RPL or RIF.

Modulation of T cell immunity in women with RPL and RIF has been a major topic in the field of clinical reproductive immunology and corticosteroids, vitamins, cytotoxic drugs, T cell activation inhibitors, biologics including cytokine inhibitors, recombinant cytokines, and immunoglobulin G, and cell therapy, have been widely investigated. In an abortion prone mouse model, mesenchymal stem cell (MSC) therapy modulated Th1 immune response to Th2 by increasing the levels of type 2 cytokines, such as IL-4, IL-6, IL-10, and GM-CSF, while decreasing IL-2, IL-12, and IFN-γ levels, locally and systemically, and led to maternal tolerance and prevention of fetal resorption (169). In LPS challenged pregnant mice, blockade of TNF-α by a soluble TNFR2-IgG1 Fc fusion protein protected fetal resorption, and placental tissue necrosis was significantly reduced (170). In high-fat-fed diabetic (HFD) NON-cNZO mice, which have higher rates of peri- and post-implantation fetal resorption, tacrolimus and metformin normalized decidual IFN-γ and progesterone receptor (PGR) expressions. In addition, these drugs increased immunophilin co-chaperone FKBP52, and co-localization of STATy and PGR, resulting in up-regulated uterine IL-11 and LIF at nidation (171). In abortion prone mice model, blocking of IL-7/IL-7R pathway by IL-7R antagonist significantly down-regulated Th17 immune responses while up-regulating Treg immunity in the decidua, and consequently, increased successful pregnancy outcome (111). In laboratory experiments using T and NK cell culture, 1,25(OH)_2_D_3_ was reported to suppress Th1/Th2 cell ratios (TNF-α/IL-10 and IFN-γ/IL-10 producing CD3^+^CD4^+^ T cell ratios) *in vitro* (172). 1,25(OH)_2_D_3_, VIPER (a TLR4 specific inhibitory peptide), and BAY11-7082 (an IKK inhibitor), effectively reduced LPS-induced pro-inflammatory cytokine production, including TNF-α and IFN-γ, through TLR4-NF-κB signaling pathway (173). The effect of 100 nM 1,25(OH)_2_D_3_ was the same as those of VIPER and BAY11-7082, suggesting vitamin D can be a potential immune regulator for women with RPL and RIF.

In clinical studies, IVIg, prednisone, TNF-blockers such as etanercept and adalimumab, prednisone, tacrolimus, and vitamin D have been reported to balance Th1/Th2 and/or Th17/Treg immunity with a varying success rate to prevent RPL (58–60, 174, 175). In a study of women with RPL (*n* = 44), IVIg treatment reduced Th1 cell levels, transcription factor expression, and type 1 cytokine levels, while increasing Th2 cell levels with the substantial decrease in Th1/Th2 cell ratios. Women with IVIg treatment had a significantly higher live birth rate (87.5%, 28 out of 32) when compared to that in controls (41.6%, 5 out of 12) (58). When TNF inhibitor was added to IVIg treatment in women with RPL and increased Th1/Th2 ratios, the success rate was increased to 71% from 54% with IVIg treatment only. However, the success rates between two regimens were not significantly different (62).

Tacrolimus is a T cell activation inhibitor. In women with RIF and tacrolimus treatment, the Th1/Th2 cell ratio predicted ART outcomes, and the pregnancy outcome was negatively correlated with Th1 cell levels in peripheral blood (60). Hydroxychloroquine, which belongs to the cytotoxic drug, was reported to decrease serum TNF-α/IL-10 ratio during the implantation window of women with RIF with down-regulation of T-bet and up-regulation of GATA-3 expression in the endometrium (176). The safety of tacrolimus and hydroxychloroquine during pregnancy has been documented (177, 178).

Vitamin D deficiency was prevalent (47.4%) among women with RPL (172), and vitamin D supplementation significantly increased Treg/Th17 cell ratios when compared to that of controls. The *in vitro* assay revealed that vitamin D up-regulated mRNA expression of vitamin D receptor and CYP24A1 as well (175). Vitamin D treatment is attractive for RPL and RIF management since it is a vitamin, and the safety for pregnancy is documented with a minimum side effect. There are few other candidate molecules or drugs possibly utilized for women with RPL or RIF. Several signal inhibitors have been utilized for the regulation of Th cells, for example, STA-21 (a dynamic STAT-3 inhibitor) and BJ-3105 (a 6-alkoxypyridin-3-ol analog) to control autoimmune diseases. However, these molecules have not been tested for the prevention of pregnancy losses (179, 180).

Criticism of a lack of double-blinded randomized trials has been made regarding therapeutics in reproductive medicine. Due to ethical, legal, and financial constraints of clinical trials for women in reproductive cycles or pregnancy, many studies have adopted either retrospective observational or controlled cohort study designs instead of a double-blinded randomized controlled trial. Even drugs often utilized for controlled ovarian hyperstimulation rarely have been validated its efficacy by double-blinded randomized trials. Over 600 drugs have been approved by the US Food and Drug Administration between 1980 to 2010, and 91% of them have been meagerly investigated for the safety of pregnant women (181). Hence, double-blinded randomized trials for pregnancy outcome or conception trials for new drugs seem a remote possibility in the near future. Until we figure out this dilemma, more data are needed under the stringent guidelines for the investigation and development of therapeutic approaches in women with T cell abnormalities.

## Conclusion

During pregnancy, a complex network of Th cells and their subsets participates in immune responses at the maternal-fetal interface. Th1, Th2, Th9, Th17, Th22, and Tfh immunities have been documented at the maternal-fetal junction but, perhaps more specific and distinctive Th subsets may present. Constraints of paternal alloantigen expression on the trophoblast cells may represent one of many mechanisms to control Th cell activation. Others may include hormonal regulations and the expression of checkpoint molecules on various immune effectors and trophoblasts, such as PD-1/PD-L1 and TIM3/Gal-9. Failures to maintain adequate Th1/Th2 and Th17/Treg cell balance are associated with RPL, RIF, and 2nd and 3rd-trimester complications. A few promising therapeutic options are available to regulate Th cell activation at the maternal-fetal junction. Further investigation of therapeutic options to control Th cell activation may shed light on treating various diseases, including cancerous disease, autoimmune diseases, and obstetrical complications.

## Author Contributions

WW and JK-K wrote the first draft of the manuscript. NS drew the figures. AG-S and JK-K reviewed and edited the manuscript. All authors contributed to the article and approved the submitted version.

## Conflict of Interest

The authors declare that the research was conducted in the absence of any commercial or financial relationships that could be construed as a potential conflict of interest.
